# Supplementary material for: Domains of Physical and Mental Workload in Health Work and Unpaid Domestic Work by Gender Division: A Study with Primary Health Care Workers in Brazil
Source: Int J Environ Res Public Health. 2022 Aug 9;19(16):9816. doi: 10.3390/ijerph19169816 (PMC9407714; doi:10.3390/ijerph19169816)
Supplement: Supplementary file 1 [file ijerph-19-09816-s001.zip › ijerph-1818034-supplementary.pdf]

Supplementary Materials

Table S1 to S7 present the complete Multivariate Linear Regression model for the Workload domains.

Table S1 - Multivariate Linear Regression for Mental Demand domain - Workload

	Non-standardized coefficients		Standardized coefficients	t	p	95% confidence interval for b	
Variables	b	standard error	Beta			Lower limit	Upper limit
Women	2,255	0,906	-0,148	-2,489	0,013	0,472	4,037
Age	-0,022	0,041	-0,043	-0,545	0,586	-0,103	0,059
Level of education	-0,015	0,254	-0,004	-0,057	0,954	-0,515	0,486
Marital status							
Single	5,438	2,741	0,474	1,984	0,048	0,045	10,831
Married	5,216	2,674	0,494	1,951	0,052	-0,046	10,477
Separate	5,528	2,750	0,331	2,010	0,045	0,116	10,939
Widowed*	0,000	-	-	-	-	-	-
Number of children	0,016	0,286	0,004	0,056	0,955	-0,546	0,578
Income	-0,872	0,485	-0,176	-1,798	0,073	-1,826	0,082
Other work parallel to that of PHC	-0,173	1,042	-0,013	-0,166	0,868	-2,225	1,878
Profession							
Doctor	7,400	1,628	0,484	4,545	0,000	4,196	10,604
Nurse	-6,873	1,596	-0,475	-4,305	<0,001	-10,014	-3,731
Nursing technician/assistant	4,554	1,721	0,356	2,646	0,009	1,167	7,940
Community health agent	3,131	1,826	0,296	1,714	0,087	-0,463	6,725
Oral health technician/assistant	2,108	2,135	0,083	0,987	0,324	-2,094	6,310
Other	1,730	2,518	0,045	0,687	0,492	-3,224	6,685
Dentist *	0,000	-	-	-	-	-	-
Length of time in the profession (years)	0,072	0,065	0,124	1,109	0,268	-0,056	0,200
Time working at PHC (years)	0,010	0,072	0,014	0,145	0,885	-0,131	0,152
Total weekly working hours	0,036	0,030	0,093	1,194	0,233	-0,023	0,096

*reference category

Table S2 - Multivariate Linear Regression for Physical Demand domain - Workload

Variables	Non-standardized coefficients		Standardized coefficients	t	*p*	95% confidence interval for b	
	b	Standard error	Beta			Lower limit	Upper limit
Women	2,215	1,008	0,135	2,198	0,029	0,232	4,198
Age	0,034	0,046	0,061	0,748	0,455	-0,056	0,124
Level of education	0,738	0,283	0,208	2,611	0,009	0,182	1,295
Marital status							
Single	2,025	3,049	0,165	0,664	0,507	-3,975	8,026
Married	3,102	2,975	0,274	1,043	0,298	-2,752	8,956
Separate	3,323	3,060	0,185	1,086	0,278	-2,698	9,344
Widowed*	0,000	-	-	-	-	-	-
Number of children	0,065	0,318	0,014	0,206	0,837	-0,560	0,691
Income	-0,869	0,539	-0,163	-1,611	0,108	-1,930	0,192
Other work parallel to that of PHC	1,102	1,160	0,077	0,950	0,343	-1,181	3,384
Profession							
Doctor	0,529	1,811	0,032	0,292	0,771	-3,036	4,093
Nurse	-0,683	1,776	-0,044	-0,384	0,701	-4,178	2,812
Nursing technician/assistant	0,910	1,915	0,066	0,475	0,635	-2,858	4,678
Community health agent	0,539	2,032	0,047	0,265	0,791	-3,460	4,537
Oral health technician/assistant	-1,688	2,376	-0,062	-0,710	0,478	-6,363	2,987
Other professionals	-3,787	2,801	-0,092	-1,352	0,177	-9,299	1,726
Dentist *	0,000	-	-	-	-	-	-
Length of time in the profession (years)	0,052	0,072	0,083	0,716	0,474	-0,090	0,194
Time working at PHC (years)	-0,079	0,080	-0,099	-0,990	0,323	-0,236	0,078
Total weekly working hours	0,005	0,034	0,011	0,141	0,888	-0,062	0,071

*reference category

Table S3 - Multivariate Linear Regression for Time Demand domain - Workload

Variables	Non-standardized coefficients		Standardized coefficients	t	*p*	95% confidence interval for b	
	b	standard error	Beta			Lower limit	Upper limit
Women	1,811	0,962	0,112	1,883	0,061	-0,082	3,705
Age	0,053	0,043	0,097	1,225	0,222	-0,032	0,139
Level of education	0,773	0,268	0,224	2,882	0,004	0,245	1,302
Marital status							
Single	5,515	2,893	0,460	1,906	0,058	-0,178	11,209
Married	4,917	2,823	0,445	1,742	0,083	-0,638	10,472
Separate	5,396	2,903	0,309	1,859	0,064	-0,317	11,109
Widowed*	0,000	-	-	-	-	-	-
Number of children	0,325	0,302	0,070	1,075	0,283	-0,270	0,920
Income	-1,196	0,512	-0,230	-2,334	0,020	-2,204	-0,188
Other work parallel to that of PHC	0,489	1,101	0,035	0,444	0,657	-1,677	2,655
Profession							
Doctor	4,164	1,722	0,258	2,418	0,016	0,775	7,552
Nurse	-3,803	1,685	-0,251	-2,256	0,025	-7,119	-0,486
Nursing technician/assistant	1,853	1,817	0,138	1,020	0,309	-1,723	5,428
Community health agent	0,397	1,928	0,036	0,206	0,837	-3,397	4,191
Oral health technician/assistant	0,971	2,254	0,036	0,431	0,667	-3,465	5,407
Other professionals	-0,589	2,658	-0,015	-0,222	0,825	-5,821	4,642
Dentist *	0,000	-	-	-	-	-	-
Length of time in the profession (years)	0,020	0,069	0,032	0,285	0,776	-0,115	0,154
Time working at PHC (years)	-0,035	0,076	-0,045	-0,460	0,646	-0,184	0,114
Total weekly working hours	0,045	0,032	0,111	1,415	0,158	-0,018	0,109

*reference category

Table S4 - Multivariate Linear Regression for Performance Domain - Workload

	Non-standardized coefficients		Standardized coefficients	t	*p*	95% confidence interval for b	
Variables	b	standard error	Beta			Lower limit	Upper limit
Women	0,880	0,958	0,056	0,919	0,359	-1,005	2,765
Age	-0,002	0,043	-0,003	-0,039	0,969	-0,087	0,084
Level of education	0,227	0,269	0,066	0,843	0,400	-0,302	0,756
Marital status							
Single	1,737	2,901	0,147	0,599	0,550	-3,970	7,445
Married	3,464	2,827	0,318	1,225	0,221	-2,100	9,028
Separate	3,407	2,907	0,198	1,172	0,242	-2,314	9,128
Widowed*	0,000	-	-	-	-	-	-
Number of children	0,213	0,302	0,047	0,706	0,481	-0,381	0,808
Income	-0,479	0,513	-0,094	-0,933	0,351	-1,490	0,531
Other work parallel to that of PHC	0,564	1,104	0,041	0,511	0,610	-1,609	2,737
Profession							
Doctor	3,189	1,721	0,203	1,853	0,065	-0,197	6,575
Nurse	-3,028	1,691	-0,202	-1,791	0,074	-6,355	0,299
Nursing technician/assistant	2,347	1,820	0,178	1,289	0,198	-1,235	5,928
Community health agent	-0,201	1,932	-0,018	-0,104	0,917	-4,003	3,602
Oral health technician/assistant	5,327	2,258	0,203	2,359	0,019	0,883	9,771
Other professionals	0,821	2,661	0,021	0,309	0,758	-4,416	6,058
Dentist *	0,000	-	-	-	-	-	-
Length of time in the profession (years)	-0,014	0,069	-0,024	-0,208	0,835	-0,150	0,121
Time working at PHC (years)	-0,023	0,077	-0,030	-0,304	0,761	-0,174	0,127
Total weekly working hours	0,006	0,032	0,015	0,192	0,848	-0,057	0,070

*reference category

Table S5 - Multivariate Linear Regression for Total Effort Level domain - Workload

Variables	Non-standardized coefficients		Standardized coefficients	t	*p*	95% confidence interval for b	
	b	standard error	Beta			Lower limit	Upper limit
Women	1,544	0,839	0,113	1,839	0,067	-0,108	3,196
Age	-0,003	0,038	-0,007	-0,089	0,929	-0,078	0,072
Level of education	0,174	0,235	0,059	0,739	0,461	-0,289	0,637
Marital status							
Single	3,572	2,542	0,348	1,405	0,161	-1,431	8,575
Married	4,152	2,478	0,440	1,676	0,095	-0,724	9,029
Separate	3,983	2,548	0,267	1,563	0,119	-1,032	8,997
Widowed*	0,000	-	-	-	-	-	-
Number of children	0,157	0,265	0,040	0,592	0,554	-0,364	0,678
Income	-0,598	0,450	-0,135	-1,330	0,184	-1,483	0,287
Other work parallel to that of PHC	0,540	0,967	0,046	0,558	0,577	-1,363	2,443
Profession							
Doctor	3,235	1,508	0,237	2,145	0,033	0,267	6,203
Nurse	-4,156	1,482	-0,319	-2,805	0,005	-7,072	-1,240
Nursing technician/assistant	2,220	1,594	0,194	1,393	0,165	-0,917	5,358
Community health Agent	0,890	1,693	0,094	0,526	0,599	-2,442	4,222
Oral health technician/assistant	1,106	1,979	0,049	0,559	0,577	-2,788	4,999
Other professionals	2,164	2,333	0,063	0,928	0,354	-2,426	6,754
Dentist *	0,000	-	-	-	-	-	-
Length of time in the profession, in years	0,000	0,060	-0,001	-0,005	0,996	-0,119	0,119
Time working at PHC (years)	-0,012	0,067	-0,018	-0,177	0,860	-0,144	0,120
Total weekly working hours	0,001	0,028	0,004	0,048	0,961	-0,054	0,057

*reference category

Table S6 - Multivariate Linear Regression for Frustration Level domain - Workload

Variables	Non-standardized coefficients		Standardized coefficients	t	*p*	95% confidence interval for b	
	b	standard error	Beta			Lower limit	Upper limit
Women	4,184	1,022	0,242	4,096	<0,001	2,174	6,195
Age (years)	-0,036	0,046	-0,061	-0,783	0,434	-0,128	0,055
Level of education	0,552	0,287	0,147	1,925	0,055	-0,012	1,116
Marital status							
Single	0,348	3,094	0,027	0,112	0,911	-5,741	6,436
Married	0,340	3,016	0,028	0,113	0,910	-5,594	6,274
Separate	0,178	3,101	0,009	0,057	0,954	-5,925	6,280
Widower*	0,000	-	-	-	-	-	-
Number of children	-0,231	0,322	-0,046	-0,716	0,474	-0,865	0,403
Income	-1,540	0,547	-0,274	-2,815	0,005	-2,617	-0,463
Other work parallel to that of PHC	0,483	1,177	0,032	0,411	0,682	-1,833	2,800
Profession							
Doctor	2,437	1,836	0,141	1,328	0,185	-1,175	6,049
Nurse	0,134	1,803	0,008	0,075	0,941	-3,414	3,683
Nursing technician/assistant	-0,226	1,940	-0,016	-0,117	0,907	-4,045	3,592
Community health agent	0,804	2,061	0,067	0,390	0,697	-3,251	4,859
Oral health technician/assistant	-5,028	2,408	-0,174	-2,088	0,038	-9,767	-0,290
Other professionals	-0,037	2,839	-0,001	-0,013	0,990	-5,623	5,550
Dentist *	0,000	-	-	-	-	-	-
Length of time in the profession, in years	0,010	0,074	0,015	0,138	0,890	-0,135	0,155
Time working at PHC (years)	0,074	0,082	0,088	0,906	0,366	-0,087	0,234
Total weekly working hours	-0,034	0,034	-0,077	-0,997	0,319	-0,102	0,033

*reference category

Table S7 - Multivariate Linear Regression for Overall/Total Workload

Variables	Non-standardized coefficients		Standardized coefficients	t	p	95% confidence interval for b	
	b	standard error	Beta			Lower limit	Upper limit
Women	1,616	0,693	0,147	2,333	0,020	0,253	2,980
Age	0,008	0,032	0,022	0,261	0,794	-0,054	0,071
Level of education	0,358	0,204	0,149	1,758	0,080	-0,043	0,760
Marital status							
Single	3,359	2,048	0,404	1,641	0,102	-0,671	7,390
Married	3,763	1,995	0,489	1,887	0,060	-0,163	7,690
Separate	4,137	2,060	0,333	2,008	0,046	0,082	8,192
Widowed*	0,000	-	-	-	-	-	-
Number of children	0,086	0,225	0,027	0,383	0,702	-0,357	0,530
Income	-0,852	0,371	-0,236	-2,296	0,022	-1,583	-0,122
Other work parallel to that of PHC	0,718	0,815	0,075	0,882	0,379	-0,885	2,322
Profession							
Doctor	3,631	1,242	0,330	2,923	0,004	1,186	6,077
Nurse	-3,456	1,231	-0,332	-2,808	0,005	-5,879	-1,033
Nursing technician/assistant	2,032	1,344	0,218	1,512	0,132	-0,614	4,678
Community health agent	1,044	1,416	0,135	0,738	0,461	-1,742	3,831
Oral health technician/assistant	1,136	1,667	0,061	0,681	0,496	-2,146	4,418
Other professionals	-0,835	1,912	-0,031	-0,437	0,663	-4,598	2,929
Dentist *	0,000	-	-	-	-	-	-
Length of time in the profession (years)	0,031	0,050	0,074	0,622	0,534	-0,067	0,129
Time working at PHC (years)	-0,044	0,055	-0,083	-0,800	0,424	-0,154	0,065
Total weekly working hours	0,004	0,023	0,016	0,190	0,849	-0,041	0,050

*reference category

Tables S8 to S14 present the complete Multivariate Linear Regression model for the Workload domains with domestic work (family involvement).

Table S8 - Multivariate Linear Regression for Mental Demand domain - Workload with domestic work (family involvement)

Variables	Non-standardized coefficients		Standardized coefficients	t	p	95% confidence interval for b	
	b	standard error	Beta			Lower limit	Upper limit
Women	1,279	1,095	0,073	1,168	0,244	-0,876	3,435
Age	-0,078	0,050	-0,129	-1,576	0,116	-0,175	0,019
Level of education	0,199	0,307	0,052	0,651	0,516	-0,404	0,803
Marital status							
Single	6,925	3,316	0,524	2,088	0,038	0,399	13,451
Married	8,403	3,232	0,690	2,600	0,010	2,043	14,763
Separate	8,318	3,324	0,432	2,502	0,013	1,777	14,859
Widowed*	0,000	-	-	-	-	-	-
Number of children	0,478	0,346	0,094	1,382	0,168	-0,202	1,158
Income	0,577	0,587	0,101	0,984	0,326	-0,577	1,732
Other work parallel to that of PHC	1,539	1,260	0,101	1,222	0,223	-0,940	4,018
Profession							
Doctor	1,601	1,967	0,091	0,814	0,417	-2,271	5,472
Nurse	-0,875	1,929	-0,052	-0,454	0,650	-4,672	2,921
Nursing technician/assistant	1,941	2,081	0,131	0,932	0,352	-2,155	6,036
Community health agent	2,567	2,207	0,210	1,163	0,246	-1,776	6,910
Oral health technician/assistant	2,933	2,537	0,103	1,156	0,248	-2,059	7,924
Other professionals	-1,709	3,043	-0,039	-0,562	0,575	-7,696	4,279
Dentist *	0,000	-	-	-	-	-	-
Length of time in the profession (years)	0,083	0,078	0,124	1,056	0,292	-0,071	0,237
Time working at PHC (years)	-0,043	0,087	-0,050	-0,495	0,621	-0,214	0,128
Total weekly working hours	-0,052	0,037	-0,114	-1,405	0,161	-0,124	0,021

*reference category

Table S9 - Multivariate Linear Regression for Physical Demand domain - Workload with domestic work (family involvement)

Variables	Non-standardized coefficients		Standardized coefficients	t	p	95% confidence interval for b	
	b	standard error	Beta			Lower limit	Upper limit
Women	1,951	1,031	0,115	1,893	0,059	-0,077	3,979
Age	-0,060	0,047	-0,104	-1,297	0,196	-0,152	0,031
Level of education	0,384	0,289	0,104	1,331	0,184	-0,184	0,952
Marital status							
Single	0,136	3,120	0,011	0,044	0,965	-6,004	6,276
Married	2,377	3,041	0,202	0,782	0,435	-3,607	8,361
Separate	1,899	3,127	0,102	0,607	0,544	-4,255	8,053
Widowed*	0,000	-	-	-	-	-	-
Number of children	0,627	0,325	0,127	1,929	0,055	-0,013	1,267
Income	-0,368	0,552	-0,067	-0,666	0,506	-1,454	0,718
Other work parallel to that of PHC	2,149	1,185	0,146	1,813	0,071	-0,183	4,481
Profession							
Doctor	-1,691	1,851	-0,100	-0,914	0,361	-5,334	1,951
Nurse	-0,223	1,815	-0,014	-0,123	0,902	-3,794	3,349
Nursing technician/assistant	0,010	1,958	0,001	0,005	0,996	-3,843	3,862
Community health agent	-0,190	2,077	-0,016	-0,091	0,927	-4,276	3,897
Oral health technician/assistant	0,290	2,411	0,010	0,120	0,904	-4,455	5,035
Other professionals	-5,526	2,862	-0,129	-1,931	0,054	-11,159	0,106
Dentist *	0,000	-	-	-	-	-	-
Length of time in the profession (years)	0,091	0,074	0,141	1,229	0,220	-0,055	0,236
Time working at PHC (years)	-0,125	0,082	-0,151	-1,520	0,129	-0,286	0,037
Total weekly working hours	-0,025	0,035	-0,057	-0,717	0,474	-0,093	0,043

*reference category

Table S10 - Multivariate Linear Regression for the Time Demand domain - Workload with domestic work (family involvement)

Variables	Non-standardized coefficients b	Non-standardized coefficients standard error	Standardized coefficients Beta	t	p	95% confidence interval for b Lower limit	95% confidence interval for b Upper limit
Women	1,338	1,013	0,083	1,322	0,187	-0,654	3,331
Age	-0,035	0,045	-0,063	-0,763	0,446	-0,124	0,054
Level of education	0,187	0,281	0,054	0,666	0,506	-0,365	0,739
Marital status							
Single	6,462	3,030	0,535	2,133	0,034	0,499	12,425
Married	7,462	2,951	0,672	2,529	0,012	1,655	13,269
Separate	7,222	3,035	0,412	2,380	0,018	1,250	13,194
Widowed*	0,000	-	-	-	-	-	-
Number of children	0,289	0,322	0,061	0,898	0,370	-0,345	0,924
Income	0,287	0,538	0,055	0,533	0,594	-0,772	1,345
Other work parallel to that of PHC	0,708	1,152	0,051	0,615	0,539	-1,558	2,975
Profession							
Doctor	-0,306	1,798	-0,019	-0,170	0,865	-3,845	3,233
Nurse	-0,773	1,761	-0,051	-0,439	0,661	-4,240	2,693
Nursing technician/assistant	-0,422	1,900	-0,031	-0,222	0,824	-4,161	3,317
Community health agent	0,333	2,015	0,030	0,165	0,869	-3,633	4,299
Oral health technician/assistant	0,777	2,316	0,030	0,335	0,738	-3,781	5,335
Other professionals	-3,416	2,780	-0,085	-1,229	0,220	-8,887	2,055
Dentist *	0,000	-	-	-	-	-	-
Length of time in the profession (years)	0,090	0,072	0,148	1,259	0,209	-0,051	0,231
Time working at PHC (years)	-0,121	0,079	-0,155	-1,523	0,129	-0,277	0,035
Total weekly working hours	-0,025	0,034	-0,061	-0,741	0,460	-0,091	0,041

*reference category

Table S11 - Multivariate Linear Regression for the Performance domain - Workload with domestic work (family involvement)

Variables	Non-standardized coefficients b	Non-standardized coefficients standard error	Standardized coefficients Beta	t	p	95% confidence interval for b Lower limit	95% confidence interval for b Upper limit
Women	0,152	0,986	0,010	0,154	0,878	-1,788	2,091
Age	-0,007	0,045	-0,012	-0,151	0,880	-0,094	0,081
Level of education	0,348	0,276	0,101	1,263	0,208	-0,194	0,891
Marital status							
Single	-0,826	2,984	-0,069	-0,277	0,782	-6,697	5,046
Married	0,922	2,908	0,084	0,317	0,751	-4,800	6,644
Separate	0,847	2,990	0,049	0,283	0,777	-5,037	6,732
Widowed*	0,000	-	-	-	-	-	-
Number of children	0,781	0,311	0,170	2,513	0,013	0,169	1,393
Income	-0,076	0,528	-0,015	-0,144	0,886	-1,115	0,963
Other work parallel to that of PHC	-0,351	1,133	-0,025	-0,310	0,757	-2,581	1,879
Profession							
Doctor	2,272	1,770	0,143	1,284	0,200	-1,211	5,755
Nurse	0,116	1,736	0,008	0,067	0,947	-3,300	3,531
Nursing technician/assistant	0,550	1,872	0,041	0,294	0,769	-3,135	4,234
Community health agent	0,279	1,986	0,025	0,141	0,888	-3,628	4,187
Oral health technician/assistant	1,447	2,282	0,056	0,634	0,527	-3,044	5,937
Other professionals	-1,416	2,737	-0,035	-0,517	0,605	-6,803	3,970
Dentist *	0,000	-	-	-	-	-	-
Length of time in the profession (years)	0,035	0,071	0,058	0,497	0,620	-0,104	0,174
Time working at PHC (years)	-0,041	0,078	-0,053	-0,521	0,603	-0,195	0,113
Total weekly working hours	-0,010	0,033	-0,024	-0,290	0,772	-0,075	0,055

*reference category

Table S12 - Multivariate Linear Regression for Total Effort Level domain - Workload with domestic work (family involvement)

	Non-standardized coefficients		Standardized coefficients	t	p	95% confidence interval for b	
Variables	B	standard error	Beta			Lower limit	Upper limit
Women	1,028	0,909	0,070	1,131	0,259	-0,761	2,818
Age	0,027	0,041	0,053	0,645	0,519	-0,054	0,107
Level of education	0,249	0,254	0,078	0,979	0,328	-0,251	0,750
Marital status							
Single	-0,269	2,753	-0,024	-0,098	0,922	-5,686	5,149
Married	1,172	2,683	0,115	0,437	0,663	-4,108	6,451
Separate	1,588	2,759	0,099	0,575	0,565	-3,842	7,017
Widowed*	0,000	-	-	-	-	-	-
Number of children	0,144	0,287	0,034	0,501	0,616	-0,421	0,708
Income	-0,247	0,487	-0,052	-0,508	0,612	-1,206	0,711
Other work parallel to that of PHC	0,679	1,046	0,053	0,650	0,516	-1,378	2,737
Profession							
Doctor	0,532	1,633	0,036	0,326	0,745	-2,681	3,746
Nurse	-1,785	1,601	-0,128	-1,114	0,266	-4,936	1,367
Nursing technician/assistant	-0,187	1,728	-0,015	-0,108	0,914	-3,586	3,213
Community health agent	1,322	1,832	0,130	0,722	0,471	-2,283	4,927
Oral health technician/assistant	2,522	2,106	0,106	1,198	0,232	-1,622	6,665
Other professionals	-1,499	2,526	-0,040	-0,594	0,553	-6,469	3,471
Dentist *	0,000	-	-	-	-	-	-
Length of time in the profession (years)	0,052	0,065	0,093	0,803	0,422	-0,076	0,180
Time working at PHC (years)	-0,035	0,072	-0,048	-0,481	0,631	-0,177	0,107
Total weekly working hours	0,024	0,030	0,064	0,786	0,432	-0,036	0,084

*reference category

Table S13 - Multivariate Linear Regression for Frustration Level domain - Workload with domestic work (family involvement)

Variables	Non-standardized coefficients		Standardized coefficients	t	p	95% confidence interval for b	
	B	standard error	Beta			Lower limit	Upper limit
Women	3,320	1,119	0,183	2,968	0,003	1,119	5,522
Age	-0,045	0,051	-0,073	-0,894	0,372	-0,145	0,054
Level of education	0,425	0,313	0,108	1,358	0,175	-0,191	1,041
Marital status							
Single	3,131	3,387	0,230	0,925	0,356	-3,533	9,795
Married	3,877	3,301	0,309	1,175	0,241	-2,618	10,372
Separate	5,004	3,394	0,252	1,474	0,141	-1,676	11,683
Widowed*	0,000	-	-	-	-	-	-
Number of children	-0,094	0,353	-0,018	-0,268	0,789	-0,789	0,600
Income	-0,216	0,599	-0,037	-0,360	0,719	-1,395	0,963
Other work parallel to that of PHC	1,499	1,286	0,095	1,165	0,245	-1,032	4,031
Profession							
Doctor	-0,401	2,009	-0,022	-0,200	0,842	-4,355	3,552
Nurse	1,453	1,970	0,084	0,737	0,461	-2,424	5,329
Nursing technician/assistant	-1,789	2,125	-0,117	-0,842	0,401	-5,971	2,393
Community health agent	0,823	2,254	0,065	0,365	0,715	-3,613	5,258
Oral health technician/assistant	-1,117	2,590	-0,038	-0,431	0,667	-6,214	3,981
Other professionals	-4,567	3,107	-0,100	-1,470	0,143	-10,681	1,547
Dentist *	0,000	-	-	-	-	-	-
Length of time in the profession (years)	0,058	0,080	0,084	0,727	0,468	-0,099	0,216
Time working at PHC (years)	0,026	0,089	0,029	0,290	0,772	-0,149	0,201
Total weekly working hours	-0,038	0,037	-0,083	-1,025	0,306	-0,112	0,035

*reference category

Table S14 – Multivariate Linear Regression for Global/Total Workload with domestic work (family involvement)

	Non-standardized coefficients		Standardized coefficients	t	p	95% confidence interval for b	
Variables	B	standard error	Beta			Lower limit	Upper limit
Women	1,418	0,787	0,115	1,801	0,073	-0,132	2,968
Age	-0,043	0,035	-0,102	-1,236	0,217	-0,112	0,026
Level of education	0,254	0,223	0,094	1,140	0,255	-0,185	0,693
Marital status							
Single	2,649	2,318	0,283	1,143	0,254	-1,914	7,212
Married	4,140	2,252	0,482	1,839	0,067	-0,292	8,572
Separate	4,148	2,332	0,303	1,778	0,076	-0,443	8,738
Widowed*	0,000	-	-	-	-	-	-
Number of children	0,517	0,260	0,138	1,992	0,047	0,006	1,028
Income	0,110	0,419	0,027	0,261	0,794	-0,716	0,935
Other work parallel to that of PHC	0,923	0,898	0,087	1,028	0,305	-0,844	2,690
Profession							
Doctor	-0,052	1,443	-0,004	-0,036	0,971	-2,892	2,787
Nurse	0,511	1,387	0,044	0,368	0,713	-2,219	3,240
Nursing technician/assistant	-0,580	1,511	-0,056	-0,384	0,701	-3,556	2,395
Community health agent	-0,173	1,601	-0,020	-0,108	0,914	-3,323	2,978
Oral health technician/assistant	0,966	1,864	0,046	0,518	0,605	-2,704	4,635
Other professionals	-3,883	2,141	-0,129	-1,813	0,071	-8,098	0,332
Dentist *	0,000	-	-	-	-	-	-
Length of time in the profession (years)	0,051	0,055	0,108	0,920	0,358	-0,058	0,160
Time working at PHC (years)	-0,031	0,062	-0,051	-0,500	0,618	-0,154	0,092
Total weekly working hours	-0,025	0,026	-0,081	-0,968	0,334	-0,076	0,026

*reference category

Tables S15, S16 and S17 present the complete Poisson Regression model for different degrees of anxiety and Tables S18, S19 and S20 the complete model for different degrees of depressive episodes.

Table S15 - Multivariate Poisson Regression for Anxiety Overall/Total

Parameter	Prevalence Ratio	95% confidence interval		p
		Lower	Upper	
Women	1,049	0,724	1,520	0,801
Level of education				
Master's/doctorate	1,095	0,611	1,962	0,760
Specialization	0,943	0,614	1,448	0,788
Complete higher education/Technology course	0,912	0,646	1,287	0,601
Incomplete higher education	0,966	0,687	1,359	0,842
Technical course	1,011	0,674	1,517	0,956
Incomplete high school/complete high school*	1			
Profession				
Other	1,159	0,582	2,309	0,675
Oral health technician/assistant	0,816	0,391	1,701	0,587
Dentist	1,169	0,625	2,184	0,625
Community health agent	1,065	0,632	1,797	0,813
Nursing Technician/assistent	0,620	0,356	1,077	0,090
Doctor	1,471	0,922	2,347	0,105
Nurse*	1			
Other work parallel to that of PHC	1,216	0,856	1,727	0,275
Marital status				
Widowed	0,423	0,065	2,766	0,369
Separate/	1,143	0,803	1,628	0,457
Divorced	0,957	0,748	1,224	0,726
Married/Consensual Union	1			
Single*				
Monthly income (m.w)	0,614	0,352	1,071	0,086
More than 8	0,615	0,365	1,037	0,068
From 4 to 8	1,019	0,768	1,352	0,896
From 2 to 4	1			
Up to 2*	0,984	0,969	1,000	0,044
Age (years)	1,013	0,985	1,041	0,375
Length of time in the profession, in years	1,020	0,992	1,049	0,164
Time working in PHC (years)	0,998	0,987	1,008	0,658
Total weekly working hours	1,065	0,970	1,169	0,187

*reference category

Table S16 - Multivariate Poisson Regression for Moderate/Severe/Complete Anxiety

Parameter	Prevalence Ratio	95% confidence interval		p
		Lower	Upper	
Women	1,226	0,705	2,132	0,470
Level of education				
Master's/doctorate	1,146	0,503	2,611	0,747
Specialization	0,881	0,477	1,626	0,685
Complete higher education/Technology course	0,824	0,493	1,377	0,461
Incomplete higher education	1,205	0,758	1,914	0,431
Technical course	1,411	0,895	2,227	0,139
Incomplete high school/complete high school*	1			
Profession				
Other	0,363	0,059	2,253	0,277
Oral health technician/assistant	0,707	0,302	1,656	0,424
Dentist	0,880	0,337	2,297	0,795
Community health agent	0,734	0,364	1,478	0,386
Nursing Technician/assistent	0,430	0,200	0,925	0,031
Doctor	1,304	0,679	2,505	0,425
Nurse*	1			
Other work parallel to that of PHC	1,158	0,659	2,033	0,610
Marital status				
Widowed	0,987	0,194	5,032	0,987
Separate/	1,081	0,635	1,842	0,774
Divorced	1,075	0,768	1,506	0,672
Married/Consensual Union	1			
Single*				
Monthly income (m.w)	0,488	0,220	1,083	0,078
More than 8	0,723	0,376	1,390	0,331
From 4 to 8	0,996	0,679	1,460	0,982
From 2 to 4	1			
Up to 2*	0,985	0,964	1,006	0,157
Age (years)	0,986	0,950	1,023	0,456
Length of time in the profession, in years	1,039	0,998	1,082	0,065
Time working in PHC (years)	0,990	0,975	1,006	0,234
Total weekly working hours	1,088	0,948	1,249	0,229

*reference category

Table S17 - Multivariate Poisson Regression for Severe/Complete Anxiety

Parameter	Prevalence Ratio	95% confidence interval		p
		Lower	Upper	
Women	1,033	0,381	2,803	0,949
Level of education				
Master's/doctorate	2,559	0,652	10,049	0,178
Specialization	1,170	0,412	3,324	0,769
Complete higher education/Technology course	1,052	0,392	2,825	0,920
Incomplete higher education	1,862	0,779	4,448	0,162
Technical course	2,516	0,993	6,379	0,052
Incomplete high school/complete high school*	1			
Profession				
Other	0,463	0,080	2,678	0,390
Oral health technician/assistant	0,275	0,054	1,398	0,120
Dentist	0,362	0,061	2,145	0,263
Community health agent	0,478	0,168	1,357	0,166
Nursing Technician/assistent	0,101	0,020	0,510	0,006
Doctor	0,943	0,297	2,998	0,921
Nurse*	1			
Other work parallel to that of PHC	1,552	0,571	4,220	0,389
Marital status				
Widowed	**	**	**	**
Separate/	1,076	0,412	2,812	0,881
Divorced	0,780	0,422	1,442	0,428
Married/Consensual Union	1			
Single*				
Monthly income (m.w)	0,214	0,049	0,926	0,039
More than 8	0,373	0,137	1,017	0,054
From 4 to 8	1,102	0,609	1,992	0,748
From 2 to 4	1			
Up to 2*	0,995	0,958	1,034	0,816
Age (years)	1,008	0,950	1,069	0,798
Length of time in the profession, in years	1,029	0,956	1,108	0,442
Time working in PHC (years)	0,981	0,946	1,016	0,280
Total weekly working hours	1,185	0,926	1,516	0,178

*reference category; ** it was not possible to estimate due to the insufficient number of cases

Table S18 - Multivariate Poisson Regression for Depressive Episodes Overall/Total

Parameter	Prevalence Ratio	95% confidence interval		p
		Lower	Upper	
Women	1,957	0,908	4,218	0,087
Level of education				
Master's/doctorate	0,961	0,294	3,143	0,948
Specialization	0,879	0,413	1,874	0,739
Complete higher education/Technology course	1,628	0,997	2,659	0,052
Incomplete higher education	1,230	0,685	2,210	0,488
Technical course	1,346	0,714	2,538	0,359
Incomplete high school/complete high school*	1			
Profession				
Other	0,637	0,106	3,837	0,623
Oral health technician/assistant	0,705	0,249	1,999	0,511
Dentist	1,240	0,441	3,485	0,683
Community health agent	0,934	0,440	1,981	0,859
Nursing Technician/assistent	0,459	0,206	1,022	0,057
Doctor	1,024	0,453	2,315	0,955
Nurse*	1			
Other work parallel to that of PHC	0,746	0,311	1,791	0,512
Marital status				
Widowed	0,525	0,088	3,149	0,481
Separate/	1,190	0,683	2,075	0,539
Divorced	0,867	0,593	1,267	0,461
Married/Consensual Union	1			
Single*				
Monthly income (m.w)	0,612	0,234	1,599	0,316
More than 8	0,458	0,204	1,032	0,060
From 4 to 8	0,785	0,480	1,284	0,335
From 2 to 4	1			
Up to 2*	0,989	0,964	1,015	0,396
Age (years)	1,037	1,000	1,076	0,048
Length of time in the profession, in years	1,010	0,969	1,053	0,642
Time working in PHC (years)	0,980	0,957	1,003	0,088
Total weekly working hours	1,101	0,939	1,292	0,234

* *reference category

Table S19 – Regressão de Poisson Multivariada para Episódios Depressivos em grau moderado/grave/completo

Parameter	Prevalence Ratio	95% confidence interval		p
		Lower	Upper	
Women	2,180	0,839	5,666	0,110
Level of education				
Master's/doctorate	1,161	0,315	4,285	0,822
Specialization	0,984	0,394	2,458	0,973
Complete higher education/Technology course	1,364	0,721	2,582	0,340
Incomplete higher education	1,161	0,564	2,390	0,685
Technical course	1,343	0,615	2,934	0,459
Incomplete high school/complete high school*	1			
Profession				
Other	**	**	**	**
Oral health technician/assistant	0,636	0,176	2,296	0,490
Dentist	1,287	0,426	3,886	0,654
Community health agent	0,868	0,342	2,206	0,767
Nursing Technician/assistent	0,347	0,109	1,106	0,074
Doctor	0,924	0,347	2,461	0,875
Nurse*	1			
Other work parallel to that of PHC	0,739	0,261	2,089	0,568
Marital status				
Widowed	**	**	**	**
Separate/	1,062	0,516	2,183	0,871
Divorced	0,805	0,510	1,272	0,353
Married/Consensual Union	1			
Single*				
Monthly income (m.w)	0,633	0,204	1,964	0,429
More than 8	0,622	0,249	1,557	0,311
From 4 to 8	0,995	0,558	1,773	0,985
From 2 to 4	1			
Up to 2*	0,988	0,957	1,021	0,483
Age (years)	0,997	0,948	1,048	0,895
Length of time in the profession, in years	1,060	1,002	1,121	0,041
Time working in PHC (years)	0,982	0,957	1,009	0,183
Total weekly working hours	1,116	0,912	1,366	0,287

*reference category; ** it was not possible to estimate due to the insufficient number of cases

Table S20 - Multivariate Poisson Regression for Severe/Complete Depressive Episodes

Parameter	Prevalence Ratio	95% confidence interval		p
		Lower	Upper	
Women	1,381	0,492	3,872	0,540
Level of education				
Master's/doctorate	4,878	1,140	20,871	0,033
Specialization	1,465	0,400	5,362	0,564
Complete higher education/Technology course	2,257	0,849	5,996	0,103
Incomplete higher education	1,595	0,481	5,285	0,445
Technical course	3,100	1,027	9,361	0,045
Incomplete high school/complete high school*	1			
Profession				
Other	**	**	**	**
Oral health technician/assistant	0,325	0,045	2,358	0,266
Dentist	0,581	0,104	3,261	0,537
Community health agent	0,735	0,226	2,391	0,609
Nursing Technician/assistent	0,122	0,014	1,038	0,054
Doctor	1,062	0,247	4,577	0,935
Nurse*	1			
Other work parallel to that of PHC	1,451	0,346	6,095	0,611
Marital status				
Widowed	**	**	**	**
Separate/	0,562	0,148	2,132	0,397
Divorced	0,677	0,362	1,266	0,222
Married/Consensual Union	1			
Single*				
Monthly income (m.w)	0,138	0,025	0,771	0,024
More than 8	0,612	0,226	1,656	0,334
From 4 to 8	0,821	0,385	1,752	0,611
From 2 to 4	1			
Up to 2*	0,995	0,941	1,052	0,853
Age (years)	0,987	0,902	1,079	0,767
Length of time in the profession, in years	1,065	0,971	1,169	0,179
Time working in PHC (years)	0,970	0,926	1,015	0,187
Total weekly working hours	1,252	0,916	1,710	0,159

*reference category
